# Pilot of a mobile money school fee payment system in rural Benin

**DOI:** 10.1371/journal.pone.0198240

**Published:** 2018-06-11

**Authors:** Claire L. Adida, Adam Chabi Bouko, Alex Verink, Ganz Chockalingam, Jennifer Burney

**Affiliations:** 1 Political Science, University of California San Diego, La Jolla, California, United States of America; 2 Centre de Promotion de la Démocratie et du Développement (CEPRODE), Cotonou, Benin; 3 California Institute for Telecommunications and Information Technology (Calit2), University of California San Diego, La Jolla, California, United States of America; 4 School of Global Policy and Strategy, University of California San Diego, La Jolla, California, United States of America; Universitat Jaume I, SPAIN

## Abstract

We present a rationale for, and results from, the pilot of a direct individual-to-institution remittance system in the context of school fee payment in rural Benin. Data confirm that school fees act as an impediment to educational attainment, and in very rural poor settings such as northern Benin, students often depend on extended family and kinship networks to pay fees. But existing remittance options are costly, in terms of fees, time, and risk. We pilot a new technology bundle in a single public high school in northeastern Benin, and evaluate its effectiveness. Here we describe the technical and institutional implementation of the project, as well as our findings from the first year of operation. We discuss takeaways and implications for scale-up.

## Introduction

Across West Africa, a key development challenge exists at the intersection of three realities. First, educational attainment is low: matriculation drops off rapidly in rural areas in high school, when fees are no longer subsidized, and this pattern is particularly remarkable for girls. Second, remittance flows—both internal and external—are a growing and critical component of income for poorer households, yet the principal mechanisms for sending money remain costly in rural sub-Saharan Africa (SSA)—in terms of time, money, and risk. Third, even with the importance of remittances, the rural poor across SSA lack access to formal banking and financial services. In spite of these conditions, mobile money—with its promise of fast, cheap, secure transactions, as well as integration of users into national and global economies—remains nascent in the regions and populations that might most benefit from its widespread adoption (e.g., [1]).

We developed a system of direct remittances for secondary school fees in Benin, West Africa, and tested it in a rural secondary school over the 2015-2016 academic year. Our innovation—which we call Prêt-à-Payer (meaning Ready-to-Pay in French)—is a technology bundle meant to address the above issues. It consists of a mobile money based platform for school fee payments, a transaction-tracking and receipting system for integration of digital and in-person transactions into one school fee payment database, and a messaging platform for communicating with students and fundraising from diaspora donors. Prêt-à-Payer (PaP) was designed to address key needs in remittances and education, while also boosting transparency around the school fee collection and budgeting process, and catalyzing mobile money use more broadly.

The core objectives of this pilot work were to assess the feasibility (both technical and institutional) of implementing this technology bundle in an extremely low-resource setting; to map in-country remittance flows for education to better understand larger-scale impact potential; to test, via an embedded experiment, the types of messaging that might work to catalyze mobile money usage, school fee payment, and donations to the school; and to obtain system usage and impact metrics for one school, which can be used to properly size a scaled-up implementation and evaluation phase. Here we describe the rural remittances environment in Benin, the PaP system, the implementation and data gathering process, results from the one-year pilot, takeaways, and plans for scale-up.

## Materials and methods

### Background and context

The solution we propose is designed for Africa’s poorest countries where a confluence of conditions holds. First, in a primarily agrarian economy, household income is scarce and seasonal [2]. Nevertheless, rural households, while cash-constrained, want to send their children to school and prioritize paying school fees; to do so, parents often rely on relatives and friends [3]. This community of funders is often non-local, and relatives either have to travel in person to pay school fees or risk sending money via a third-party.

Observational studies drawing from detailed survey data have shown that these remittances—typically from relatives who have emigrated or relocated to urban areas for employment in non-agricultural sectors of the economy—are critically important for livelihoods more broadly in rural sub-Saharan Africa, both due to overall poverty levels and vulnerability [4–6], and because the formal banking and financial sectors are underdeveloped. [7]. In the absence of formal financial services, remittances have been shown to improve access to public services [8], provide insurance against income shocks [9], and even stimulate agricultural productivity [10].

And yet, remittances are not working as well as they should. Three obstacles limit the effectiveness of remittances as an informal insurance scheme in this context –transaction costs, control, and cultural norms. The first has been well-documented: the cost of sending remittances can be prohibitively high. The World Bank estimates that, globally, sending remittances costs an average 7.32% of the amount sent, and this share increases to 9.42% for sub-Saharan Africa [11]. Second, even within the same household, individuals may hold different preferences [12]: this matters because remittance-senders and remittance-recipients may diverge in how they would like the money spent, and absent a control mechanism, senders may remit less than they otherwise would. Finally, migrants who have to travel back home to remit may face added pressure to share the wealth [13, 14]; as a result, they may prefer not to send money home at all. Remittances thus offer a potential solution, but they also do not entirely fulfill their development promise.

A more recent wave of experimental (randomized control trial) studies on remittances have shed light on the possibility for mobile money, with its reduced transaction costs and technological flexibility, to address some of these inefficiencies. An field experiment using an encouragement design in rural Mozambique showed that access to mobile money promoted increased urban-rural remittance flows over meaningful time scales (3 years at publication). Importantly, these remittance flows translated into reduced vulnerability to hunger shocks for receiving households, along with the ability to pay some school-related expenses (books and uniforms) even in the face of shocks. [15]. A similar experimental study in Bangladesh also resulted in increased remittance flows to rural households, reduced lean-season vulnerability, increased savings and schoolings, and decreased child labor. [16]

Two other experiments that did not directly test the potential for mobile money based remittances to address the control and cultural norms issues, nevertheless offer suggestive findings in favor of such systems: A study in El Salvador showed that when remittance-senders are given the option to send their remittances via joint bank accounts (in other words, when they are given greater control over how the money is used), they send more money home [17]. Finally, a mobile money based savings experiment with farmers in Mozambique found that interventions with groups of farmers in the same network reduced pressure to “share the wealth” and resulted in greater savings by all farmers involved. [18] These two studies suggesting that mobile money accounts could potentially be configured to alleviate the control issues and cultural norm pressures that presently impede optimal remittance flows.

**Fig 1:**
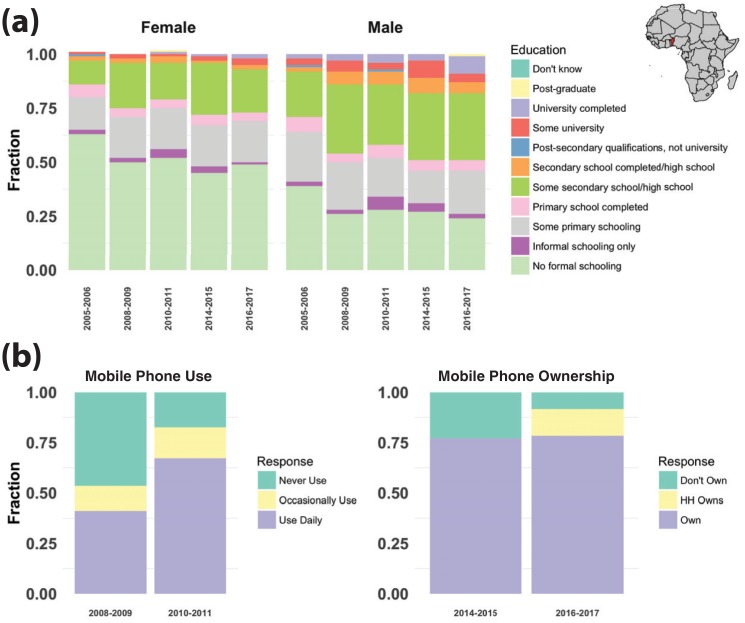
Educational attainment and mobile phone usage/ownership in Benin, 2005-2017. (a) Educational Attainment by Gender. Educational attainment has risen over the past decade in Benin, but at different rates for men and women, particularly for secondary school. (b) Mobile phone usage (Afrobarometer rounds 3 & 4) and ownership (Afrobarometer rounds 6 & 7) in Benin. Access to mobile technology has risen rapidly over the past decade. Location of Benin shown in inset (red). Responses from Afrobarometer rounds 3-7 (2005-2017) [1].

However promising for remittances, the impact of mobile money in most of sub-Saharan Africa has been limited [19, 20] by the fact that uptake has been slow [21] in those countries that have most to gain from its widespread adoption. This is certainly the case in our study country, Benin. At the national level, education rates remain low, despite recent policies to address the issue (Fig 1(a)). In October 2006, Benin implemented part of the promise of its 1990 Constitution tasking the State with ensuring free public education and making primary school compulsory (Articles 12 and 13). On October 14, the Council of Ministers decreed free public schooling for all, but this was implemented only at the primary level. As of 2010, responding to a dire gender disparity in secondary education, the government has gradually exempted female students from paying school fees for the first cycle of secondary education as well.

The country’s efforts to expand public education have paid off to some extent. Indeed, the proportion of Beninois who have completed at least primary education rose from 29% in 2005 to 43% in 2017. But gains have been slower in secondary education, where school fees come into effect. Six percent had completed at least secondary education in 2005; this rose to only 13% in 2017 [1]. Disconcertingly, the disparity in educational attainment rates between men and women has widened over time (Fig 1(a)). Rural families surveyed for an unrelated study nevertheless express a strong desire to send children to school, but report deep financial constraints. [22] Family members and friends in Benin who want to pay for school fees for children living elsewhere have limited options for sending remittances—in-person travel, expensive or inconvenient standard bank transfers, taxi or bus driver. These options suffer from the inefficiencies described above, and are costly for the families and the schools: families incur significant risks and transaction costs, and schools often take months to collect their fees.

At the same time, as it has across sub-Saharan Africa, mobile phone usage and ownership have risen rapidly in Benin (Fig 1(b)). In 2008 (Afrobarometer Round 4), just over half of the surveyed respondents nationally reported ever *using* a mobile phone; by 2017, over 91% of respondents reported that they or someone else in their household *owned* a mobile phone.

Our solution thus leverages two facts about rural households in Benin to make remittances work better: rural households want to send their children to school, and they own cell phones. A mobile money school-fee payment system thus promises to solve the above-challenges: rural households already have access to mobile phones; remittance-senders have control over the use of their remittance; and mobile money transactions are private.

### Technology description

Our product is a bundle of three technologies, developed between May and October 2015 and refined from October 2015 to October 2016, meant to address key needs in remittance networks and rural education, and to catalyze mobile money uptake and use more broadly. These components, their rationale, and details of their development are described below:

**A mobile money platform**: The individual-to-institution capability was developed in conjunction with MTN, our mobile provider partner, to interface with their existing mobile money platform, called ‘MoMo’. It is a USSD menu-based interface, designed by our team, for use on feature phones. This is a critical piece of our intervention, as we are operating in a region where feature phones—but not smartphones—are prevalent. Donors access the school fee payment menu, select a destination school, input a unique student ID code (that we generate for students), and select the amount and purpose of the transfer (e.g., school fee payment, donation to school, etc.). On the receiving end, the school has an account in the name of the principal and accountant; the accountant can cash out the account as needed, up to twice per week, to transfer the money into its official institutional bank account.

**Simple transaction-tracking software**: Most record-keeping in rural schools is done by hand, but school administrators want to know more about who is paying, and when, to better budget during the school year and try to raise funds for students in need. In addition, local parent associations—who work directly with their local school administrators to craft the school annual budget—lament the lack of standardized and timely information on school finances. We access the transaction stream from the school fee payment platform via a SOAP-based API provided by MTN at a fixed time interval (once per minute), parse it into fields, and send confirmation receipts by SMS to the school accountant. In addition, transaction information is entered automatically into a web-based student payment database. The accountant can also manually add in-person payments to the student payment database. At present this transaction-tracking platform is hosted on Amazon Cloud Servers and accessed via modem over the MTN cellular network in Benin. The student payment database was designed for easy data entry by school administrators (normally the accountant) and multi-function use. Data can be sorted and viewed by student, by date, by class. Stored fields include the student ID, class, year, a contact phone number if the student has one, as well as any information from payment transactions (e.g., donor phone number). The initial design of the web-based system included bells and whistles, such as dynamic searches, which slowed down the download speeds of the web pages. Hence we scaled back on some of the features to make the pages lightweight. In the final version, each page was less than a few kilobytes in size and allowed for fast access via the MTN modems.

**SMS-based messaging platform**: At present, any communication between school administrators and students/parents/donors (those who pay school fees on behalf of students) is done by word of mouth through the school’s parent association, and is limited in scope and efficiency. The messaging platform uses MTN and Moov (the other main network in the pilot region) modems to distribute mass SMS messages to subsets of the greater school community. This could be an announcement by the administration about a meeting, school closure, or exam date to the entire student body, or a distinct and targeted fundraising appeal to donors who have used the mobile money based payment system. The messaging platform is integrated with the student and payment database, and designed for easy use by administrators with low technological proficiency.

### Implementation

The pilot implemented the innovation in Kalalé, Benin, a commune of approximately 170,000 people, 80 to 90% of whom depend on agriculture for their livelihoods, and where the median resident survives on less than $1.90 a day [22]. We chose Benin in part because our team already had extensive fieldwork experience, a network of relations, and a history of successful interventions previously implemented in collaboration with a local community-based development organization there. In June 2014, our team traveled to Benin to introduce the idea to the pilot school and to discuss our innovation with stakeholders at all levels—the school administration, students, the parent-teacher organization, the local community-based development organization, commune administrators, members of the diaspora within Benin (who pay school fees for dozens of students), the two main cell phone companies, and the Ministries of Secondary Education and of Telecommunication. Our proposal was met with significant enthusiasm and enjoyed buy-in from local stakeholders to the highest levels of government. Similar success was achieved with the mobile money division of the country’s largest cell phone company.

The public secondary school in Kalalé serves a total 1,870 students, and is similar in size to regional secondary schools around the country. At the time we implemented our pilot, school fees were covered only for girls from grades 6 to 9. All students had to pay their annual school fees of CFA12,000 (approximately $25) from grades 10 to 12.

A key goal of this pilot study was to catalyze mobile money use. Accordingly, we intentionally chose a very remote and low-resource setting for pilot testing of this project. Mobile money use was predictably very low, and this meant that implementation by necessity required much more than simply advertising the existence of a new product and waiting to see if it would be used. CEPRODE (the Centre de Promotion de la Démocratie et du Développement), a non-profit non-governmental association, led the implementation in the field, which consisted of the following steps.

- **Stakeholder meetings**: Our team first conducted a set of meetings with key stakeholders in June 2014 to assess feasibility and support for the project. We followed up with all key stakeholders at the start of the pilot (August 2015), including local government officials, the Ministry of Education, MTN, school administrators, and the local parent association. All reaffirmed support, with the full meeting of the parent association providing key feedback on the desirability for this capacity along the dimensions discussed above.
- **Student survey**: At the start of the 2015 school year, as administrators were organizing students into classes and gathering information from them, we interviewed each student over the age of 13 in the Kalalé school to find out where they came from (many originate from outside the main village), how they usually payed their school fees, difficulties they had encountered, etc… Most important, we gathered contact information for their donors—those who contribute to their education costs. Overall we gathered data for 629 students (this represented all consenting students grades 9 through 12), with contact information from 662 donors around the country. A majority of donors were parents of students, though there were also aunts/uncles, friends, and former teachers/mentors. We obtained contact information of donors from the students themselves.
- **Donor outreach**: The CEPRODE team attempted to contact all donors, subject to IRB restrictions which limited our total number of attempts to 3; the team successfully reached and interviewed 241 donors. Donors were informed about the new platform available for Kalalé students, told how they could enroll in and access the platform on their own, and invited to registration events if they wanted help enrolling. We also administered a small survey of donors.
- **Embedded donor survey experiment**: One objective of this pilot study is to improve our understanding of the kinds of messages that effectively induce mobile money uptake and donations to the school. To do so, we tested two possible interventions. First, grounded in the social science literature on network and peer effects [23], we tested whether telling subjects that they were part of a larger community of mobile-money donors to the school would induce greater adoption of the technology (*Peer effect*). Second, following other impact evaluations testing the determinants of mobile-money uptake [15], we tested the effectiveness of a matching gift incentive (*Altruism*). Finally, we combined both messages to test whether a more forceful intervention highlighting both peer effects and matching incentives might induce technology adoption and donations (*Peer effect + Altruism*). Donors were randomly assigned to either the control condition, which received no particular additional message, the *Peer effect* condition, the *Altruism* condition, or the combined *Peer effect+Altruism* condition:
  - Peer effect: “We want you to know that we are reaching out to an entire community of school-fee payers like yourself. There are 2,000 of you, which means that if you all choose to use this system, we will successfully have created a community of mobile money users in Kalalé.”
  - Altruism: “In addition, to help you in this process, we are offering to donate money to the CEG-Kalalé if you enroll and pay your student’s school fee in full by the payment deadline. You can choose for this donation to go either to the general school fund, the school library, the campus enclosure, or the computer center. Which would you prefer?”
  - Peer effect + Altruism: “We want you to know that we are reaching out to an entire community of school-fee payers like yourself. There are 2,000 of you, which means that if you all choose to use this system, we will successfully have created a community of mobile money users in Kalalé. In addition, to help you in this process, we are offering to donate money to the CEG-Kalalé if you enroll and pay your student’s school fee in full by the payment deadline. You can choose for this donation to go either to the general school fund, the school library, the campus enclosure, or the computer center. Which would you prefer?”
- **Registration events**: We hosted a series of registration events between October and December 2015, in and around the pilot region, to help the donors we had contacted (and others who were interested) register for MTN mobile money and learn, in person, how to make a payment with the system. We purchased the SIM cards for those who did not have them, and also offered a credit incentive for coming. 236 of the 241 contacted donors came to the registration events, along with approximately 125 others who had heard by word of mouth, but had not been a part of our survey sampling frame.
- **Monitoring of transactions**: With the system in place, we then monitored transactions remotely, and our CEPRODE team worked with the school accountant to make sure he could enter the data he wanted, access information, cash out the school’s account correctly, etc.
- **Testing of messaging platform for communication and fundraising**: Due to challenges with the MTN platform—which migrated in December 2015—we only tested the messaging platform twice: once to advertise the fall fee payment deadline and once to announce the new access code for the migrated MTN menu.

Human subjects approval for all steps listed above was granted by the UC San Diego Institutional Review Board, #150857S, prior to implementation. A Pre-Analysis Plan for the project was also registered with EGAP (http://egap.org/registration/1426).

## Results

**Fig 2:**
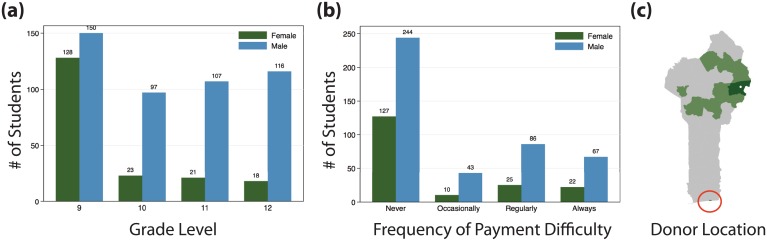
Demographics of the pilot school student body. (a) Student Enrollment by Grade and Gender. Enrollment for boys and girls is nearly equal in 9th grade (troisième), when schooling is still free for girls (subsidized by government). The ratio of girls to boys drops precipitously with the transition to high school and the end of subsidies. (b) Self-Reported School Fee Payment Difficulty by Grade and Gender. 42% of enrolled boys and 30% of enrolled girls report some level of payment difficulty, but since school fees are paid for 9th graders, the girls reporting payment difficulties are exclusively from upper grades (10th-12th), with over 90% of those reporting some level of difficulty. (c) Locations of individuals (donors) paying school fees for students in green; study school location marked with white point. Payers are dispersed throughout the country, including in the largest city, Cotonou (red circle), around 600km away. At least one payer was also reported from Nigeria, Niger, and Germany.

Our student survey helped us understand baseline conditions for school fee payment in rural areas. We confirmed a large drop of more than 80% in female enrollment between 9th grade (which has 100% government subsidy for girls) and 10th grade (no subsidy). Male enrollment also declines by 35% between the two grades, giving an estimate for the baseline retention rate (students must pass an exam to enter 10th grade). 253 students, or 40% of our student sample, reported that paying school fees is currently or has recently been difficult (Fig 2).

**Fig 3:**
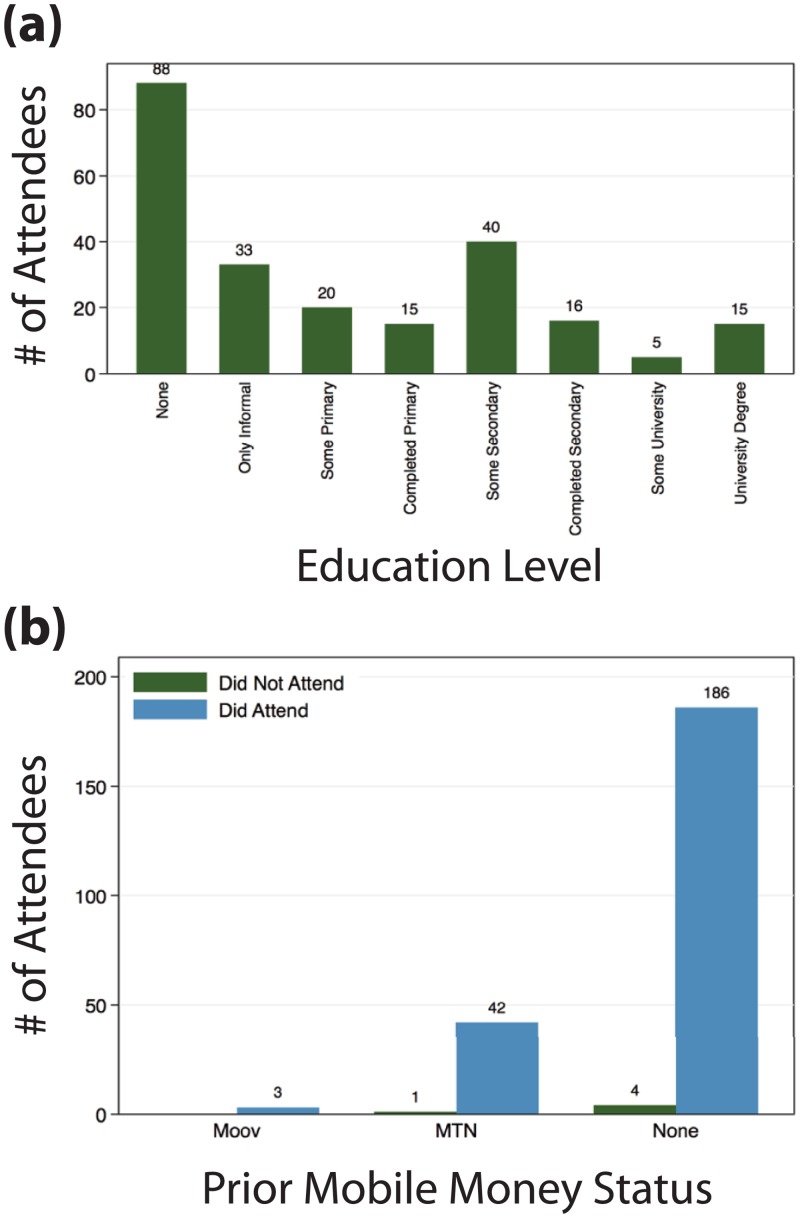
Registration workshop attendance by putative donors. Of the list of 662 donors generated by our student survey, we were able to reach and survey 241 donors, and invited all to registration events to learn to use the PaP system and register for MTN mobile money accounts. Of the 241, 236 attended registration events; this attendance spanned (a) donor education levels, including a majority with no formal schooling, and (b) prior mobile money experience, where the majority (186) had no prior experience with digital payments.

A key question for digital financial services aimed at the unbanked is whether or not they reach the target demographic and indeed enable inclusion of those who might not otherwise use such platforms. To assess this, we tracked who, of the contacted donors, actually showed up at a registration event to learn about the system, open an account, and learn to use mobile money both to pay school fees and in general. The existence of personalized enrollment events seemed to be very popular, with almost everyone we contacted attending an event. Moreover, we found that the PaP system was appealing to donors, and there is a high level of interest in mobile money for school fee payments across donor demographics. Of the 241 donors reached by phone and surveyed, only 19% reported having a mobile money account, most with MTN (the partner in this project), and a few with Moov (the other leading network in Benin). These donors nevertheless showed up to events at very high rates. Most noteworthy is that 186 individuals whom we contacted and who had no prior mobile money experience showed up to register, along with an additional 125 individuals who were not part of our original donor list but who heard about the events and came. A large majority of individuals signing up for mobile money had little to no formal education (Fig 3). This indicates that such targeting and outreach strategies indeed reach the intended audiences.

**Fig 4:**
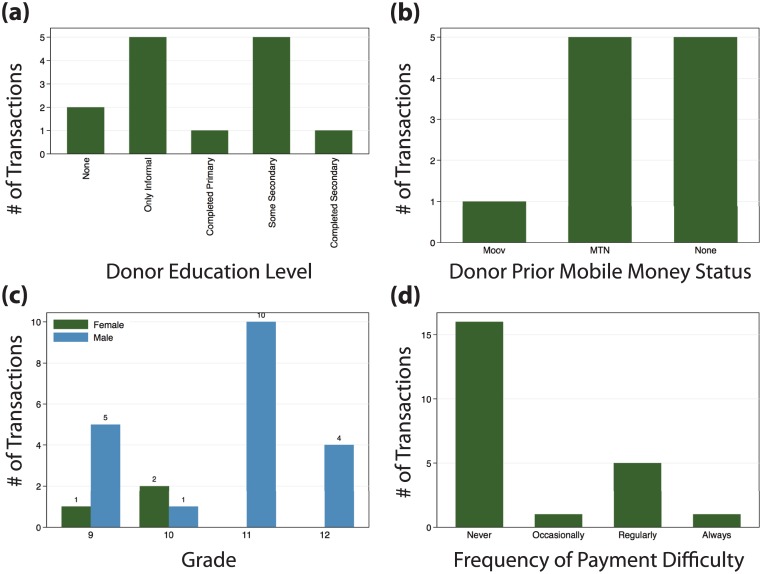
Transactions. Donors made 23 payments on behalf of 18 students using the PaP system when it was active. Although numbers are too small to assess statistical differences, payments were distributed across: (a) donor education level, (b) donor prior mobile money experience, (c) student gender and grade, and (d) prior payment difficulties as reported by the students. The number of transactions linked to donors is lower than the total, as 9 payments came from donors who were not reached in the donor survey, or not listed in the original student-derived roster of donors.

In the pilot phase, 15 donors made 23 transfers on behalf of 18 students. School fees in Kalalé are 12,000FCFA per year (approximately $25). The school received a total of 165,000FCFA via the PaP system out of an anticipated 6,000,000FCFA in fees (based on the number of students and subsidy levels). This is around 3% in terms of both total amount and number of students who received transfers. Most donors paying fees electronically paid in full. Transfers were somewhat dispersed across grades; while overall numbers are small, no girls in the upper two grades received transfers. While 16 of the transfers made through Prêt-à-Payer were for students who reported never having trouble paying school fees, 7 transfers were received by students who reported difficulty paying fees, indicating that the convenience factor may be appealing for both ends of the socioeconomic spectrum (Fig 4a and 4b).

On the donor end, 6 transfers were made by individuals with previous mobile money experience, while 5 were made by new users. Note that we cannot know the previous experience of the other donors, as they were not part of the original donor list provided by students. We find it especially encouraging that 9 of the transfers were made by individuals who we did not contact by phone, who found out about the system on their own, enrolled, and used it (Fig 4c and 4d). We are also unable to track whether new users who made transactions with the system used their new mobile money capacity more broadly for other transactions, but working with providers to track this in the future is a key priority for testing whether specific digital financial services in fact catalyze more general transformations.

**Fig 5:**
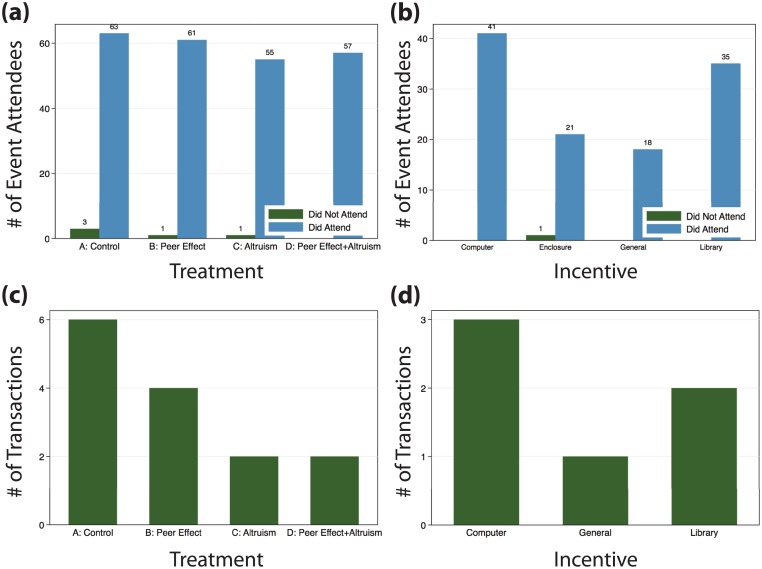
Experiment. We conducted an embedded experiment when we surveyed donors to see what types of prompts (peer effects, a matching incentive, or both) might incentivize them to use the PaP system to pay school fees by an earlier deadline. Donor attendance at registration events was almost 100% and thus (a) independent of treatment. However, (b) donors overall strongly preferred to use matching funds for a computer or library, not general funds or improvements to school grounds. At the transaction level, numbers are again too small to assess statistical differences, but seem independent of (c) experimental treatment or (d) a donor’s particular preference for how to use matching funds.

Our small embedded survey experiments with donors did not reveal any statistically significant differences (two-sided difference of means tests) in either attending an event or making a transaction by treatment status (Fig 5a and 5c). When we asked donors where they would prefer us to make a small matching donation if they paid school fees in full with the system, they overwhelmingly preferred donations to support the building of a library and purchase of computers versus general operating expenses or building of an enclosure around school grounds (Fig 5b). There is no evidence that this choice is correlated with use of the PaP system.

Towards the end of the school year we were able to collect transaction records from the school accountant, who had used the transaction tracking software to log in-person transactions as well. His records showed 171 in-person transactions in addition to the PaP payments, meaning that almost 12% of recorded transactions were through mobile money. While this number is indeed encouraging, the denominator indicates a combination of non-payment and/or unrecorded transactions. At best this represents only a third of anticipated transactions, and provides further evidence for the poverty at the institutional level: schools often do not receive payment from all students, but only dismiss them at the end of the year, when they must pay to sit for exams or leave. We had planned to conduct fundraising experiments with diaspora communities during this pre-exam period, but due to MTN’s technical difficulties internal to their own mobile wallet platform migration (discussed below), we were unable to do so. The sparse transaction record additionally highlights the need for better transaction tracking and transparency at the school level.

## Discussion

A number of factors contributed to the low uptake and use we reported above:

1. Technological constraints: Shortly after launching PaP, MTN migrated its entire mobile money system, breaking our API and registering transactions with incorrect error codes. As we waited and worked toward a resolution (a process that took approximately 9 months), we downscaled our efforts to encourage individuals to use the system. This was a main contributor to relatively low uptake and use: most of the transactions described occurred prior to the system migration.
2. Institutional constraints: MTN offered only two types of accounts at the time, individual and corporate, with cash-out limitations on each. Neither existing structure was appropriate for a school: at present, official school accounts are largely held through the national agricultural bank (CLCAM), which cannot be directly linked to MTN mobile money accounts. The inability to link coupled with the inability to cash out frequently made it more difficult to obtain buy-in from school administration. Our team overcame this problem in part by convincing MTN to allow for more frequent cash-out from the accountant and to train and post an additional mobile money cash-out agent with the ability to serve the school account permanently in our pilot village.
3. Structural constraints: Our donor data shows that the network of school fee donors was geographically diffuse, confirming the need for many donors to travel or send money to pay school fees. However, fewer donors than anticipated lived in areas with existing mobile money infrastructure—registration and cash-in agents. Thus, the demand for a platform like PaP may be greater than is currently reflected by the number of transactions in the pilot, as many donors were unable to add funds to their mobile money accounts in their locality. This is a symptom of the “chicken-and-egg trap” that is common with mobile money expansion [21].
4. Regulatory environment: There is currently no framework in Benin for schools to legally process mobile payments. The Ministry of Education gave us its support for the pilot, but school administrators were (rightfully) nervous about establishing a payment structure without clear guidelines analogous to those for in-person payments. Moreover, although this was a pilot, there was already concern from both users and administrators about preferential treatment of MTN over other providers, as Benin has an active and competitive mobile provider market (http://arcep.bj/operateurs-de-reseaux-de-telephonie/). We met with representatives from the Ministry of Education at local, regional, and national levels to secure approval for the pilot project.

We draw from this pilot and its results the following takeaways. First, a mobile phone provider may not be the ideal partner in this endeavor, especially in a competitive market. Instead, any scale-up should plan to work directly with a mobile money payment platform to develop a provider-neutral solution that telecommunication companies can easily adopt. Second, the institution receiving the payment—here, the school—must either have easy access to cash-out opportunities, or to a mobile money wallet that is approved by the regulatory and legal structure, preferably a payment system linked directly with the school’s bank account. Third, the “chicken-and-egg” problem suggests the need for a platform like PaP to be supported by a simultaneous expansion of capacity for the mobile money platform in order to satisfy the demand generated. Finally, the obstacles to uptake on the demand side are important but not prohibitive. The incentives we offered to join a registration event were small, and word-of-mouth easily expanded our reach. Nevertheless our on-the-ground team put in an enormous effort to teach stakeholders about the system, help them enroll and understand the benefits of digital payments, and help them make payments and trust the electronic receipting. Future research should focus on the true extent of nudging needed in different contexts to catalyze uptake.

## Conclusion

PaP is an innovative technological and institutional solution that is incentive-compatible: government officials want higher educational attainment rates; students in poor areas often rely on donors in different locations for school fee payment; parents and donors lament the cost and risk of existing methods of payment; school administrators want to be paid earlier for easier budgeting, and want the opportunity to fundraise for school improvements or scholarships for students in need; local parent associations want greater budgeting transparency; and mobile money providers want to find a foothold into the market at large, but particularly into rural and poor households.

Our pilot demonstrated the potential our solution offers, and highlighted two key obstacles: telecommunication companies may not be the right partner for this type of intervention; and the recipient institution must have access to a mobile money wallet that is in line with regulatory and legal structures. The good news, however, is that obstacles to uptake did not come from the demand-side.
